# The Dilemma: Whether to Proceed or Postpone a Patient With Pyrexia at Induction of Anesthesia for Coronary Artery Bypass Graft Surgery

**DOI:** 10.7759/cureus.20343

**Published:** 2021-12-11

**Authors:** Sean R Bennett, Muneeb Alnouri, Jose A Fernandez

**Affiliations:** 1 Anesthesiology, Cardiac and Critical Care, King Saud Bin Abdulaziz University for Health Sciences, Jeddah, SAU

**Keywords:** autobiographical case report, anesthesia, gram negative cultures, right ventricular compression, right ventricular dysfunction, cardiac surgery, pyrexia

## Abstract

Routine surgery may be postponed if a patient has high white blood cells (WBC) and/or pyrexia. However, postponement carries the risk of myocardial ischaemia or infarction in a patient having coronary artery bypass graft (CABG) surgery. Our case raises this dilemma in a high-risk patient that was further compromised by acute right ventricular (RV) dysfunction. A 51-year-old diabetic with end-stage renal failure, chest pain, and a recent non-ST elevation myocardial infarction (NSTEMI) who had previously refused surgery now presented for urgent CABG. During central line insertion, he started shivering and stated that he felt cold. His temperature was not measured pre-intubation, but he felt warm to the touch with no chest pain. Blood pressure (BP) 190/80 mmHg and HR 110 bpm. Iv glyceryl nitrate (GTN) and fentanyl controlled the BP. Cerebral oximetry was used to measure brain regional saturation (rSO_2_) with probes placed on the forehead pre-induction. Post-intubation his temperature was 38.1°C, end-tidal carbon dioxide (EtCO_2_) 9.2 kPa, heart rate (HR) 120 bpm. His recent NSTEMI and surgical referral two years previously meant that his ischaemic risk was high, and we decided to proceed with the surgery. During the internal mammary artery (IMA) harvesting and use of a retractor (IMAR), there was a steady fall in the rSO_2_ readings along with hypotension and an increase in central venous pressure (CVP) becoming critical after 60 minutes. At this point, the patient went onto cardiopulmonary bypass (CPB). The patient required triple vasoactive support to wean off CPB. In the intensive care unit (ICU), he required immediate support for RV failure, including nitric oxide. The next day, the patient grew Gram-negative blood cultures. In hindsight, we should have checked his temperature before induction and postponed or postponed post-induction. Regarding the IMAR or any retractor, the operating team will pay much closer attention to any haemodynamic changes resulting from their use and act accordingly.

## Introduction

Typically, routine major surgery is postponed if a patient has signs of impeding or actual infection, as indicated by a raised WBC count and/or pyrexia. The reasons are not wanting to complicate recovery with community-acquired infection, the risk of causing surgical site infection, and uncertainty as to the severity of an impending infection. Coronary artery bypass graft (CABG) surgery has the added dimension of the risk of myocardial infarction in the non-revascularized patient caused by the postponement. This case was high-risk due to recent myocardial infarction and dialysis and raised the dilemma of 'proceed or postpone' when showing the first signs of infection (shivering, pyrexia, and tachycardia) at the time of induction of anesthesia. The preoperative vital signs were normal. The case was further compromised by acute RV dysfunction. The differential diagnosis of these signs is discussed, along with how this patient could have been managed differently. 

## Case presentation

A 51-year-old male diabetic with end-stage renal failure requiring dialysis presented to our cardiac centre with chest pain. He was diagnosed with a non-ST elevation myocardial infarction (NSTEMI). Troponin 583pg/ml (>x10 normal) and his angiogram showed diffuse coronary disease. Two years earlier, he had been admitted with angina and offered CABG surgery, but he refused. On this admission, his chest pain resolved with treatment, his troponin I recovered, and he was dialysed thrice to optimize his condition. Again, he was told, coronary stenting was not an option, and this time he agreed to CABG surgery. He had normal left ventricular (LV); and RV systolic function, tricuspid annular plane systolic excursion (TAPSE) was 25mm (normal >15mm). The coronary disease was confined to the left circulation. After dialysis the day before surgery, his WBC was 7,100 cells/µl with a neutrophil count of 4,100 cells/µl, and on the morning of surgery, the ward recorded a body temperature of 36.5°C. On arrival in the operating room, he was given 1 mg midazolam iv before insertion of a right brachial arterial line. The anesthetist proceeded to place a right internal jugular triple lumen line using local anesthesia as per normal practice. At this time, the patient started shivering. He had not been given any medication that could cause hyperthermia. He said he felt cold but was warm to the touch and had no chest pain. A warming blanket was put on him. His BP was 190/80 mmHg and his HR 110 bpm. He was given iv GTN and fentanyl, which controlled the BP, but his HR increased. He continued to shiver, and the anesthetist decided to induce anesthesia and place the central line after induction. Post-intubation, the nasopharyngeal temperature showed 37.9°C and soon after 38.1°C. End-tidal carbon dioxide (EtCO_2_) was 69 mmHg/9.2 kPa; HR was 120 bpm. Transesophageal echo (TEE) showed normal LV and RV function. At this point, there was very low exposure to sevoflurane, and his temperature did not rise, further ruling out malignant hyperthermia. His haemodynamics were controlled with GTN and metoprolol 1mg. The surgeons were informed, and the decision was made to continue with the surgery, given his presentation. Antibiotics of cefazolin 2g and gentamicin 400mg were given. Anesthesia was fentanyl, cisatracurium, and sevoflurane.

**Figure 1:**
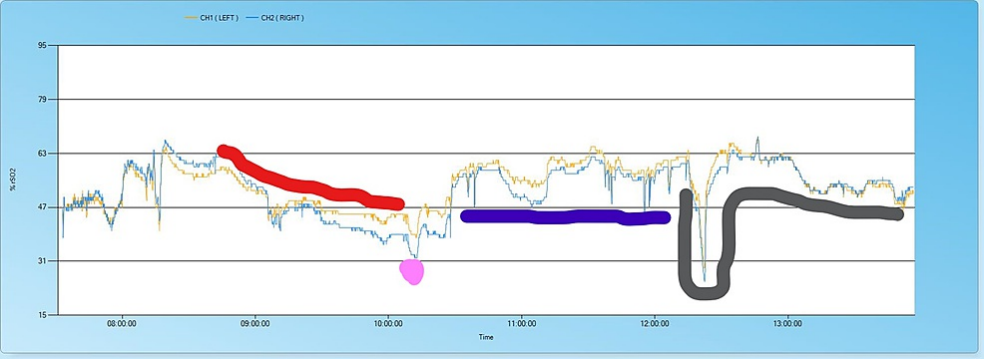
Cerebral Oximetry recording from left and right frontal cortex during the surgery. Colours label the intraoperative events. The red line indicates a fall in cerebral oximetry during mammary harvesting. Purple dot shows an acute drop in cerebral oximetry to 32%. The blue line indicates cardiopulmonary bypass, during which cerebral oximetry is maintained above the baseline of 47%. The black line shows acute fall below 30% at the first attempt to wean cardiopulmonary bypass.

During left IMA harvesting, his rSO_2_ started to drop from 63% towards 40% Figure 1.

**Figure 2:**
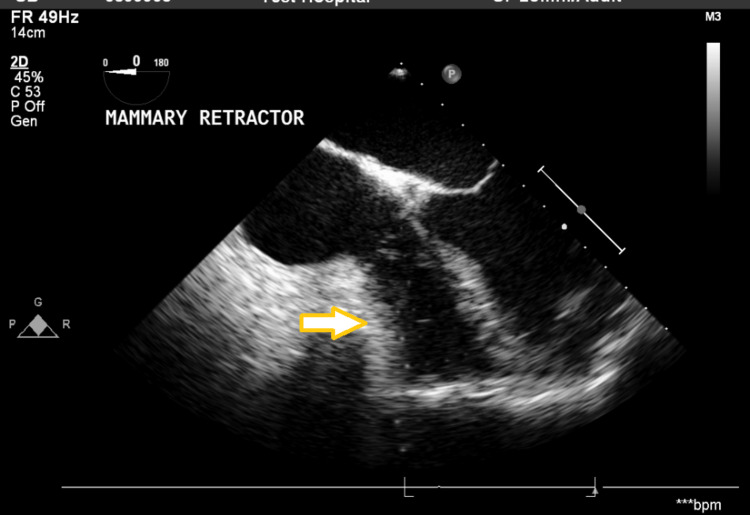
Transoesophageal echo in the four chamber view showing compression of the Right Ventricle by the mammary retractor (arrow).

His central venous pressure (CVP) increased from 12 to 16 cmH_2_O. Having checked other causes, it appeared that the IMAR was affecting RV performance. Figure 2 shows the echo image of the IMAR impinging on the RV.

**Figure 3:**
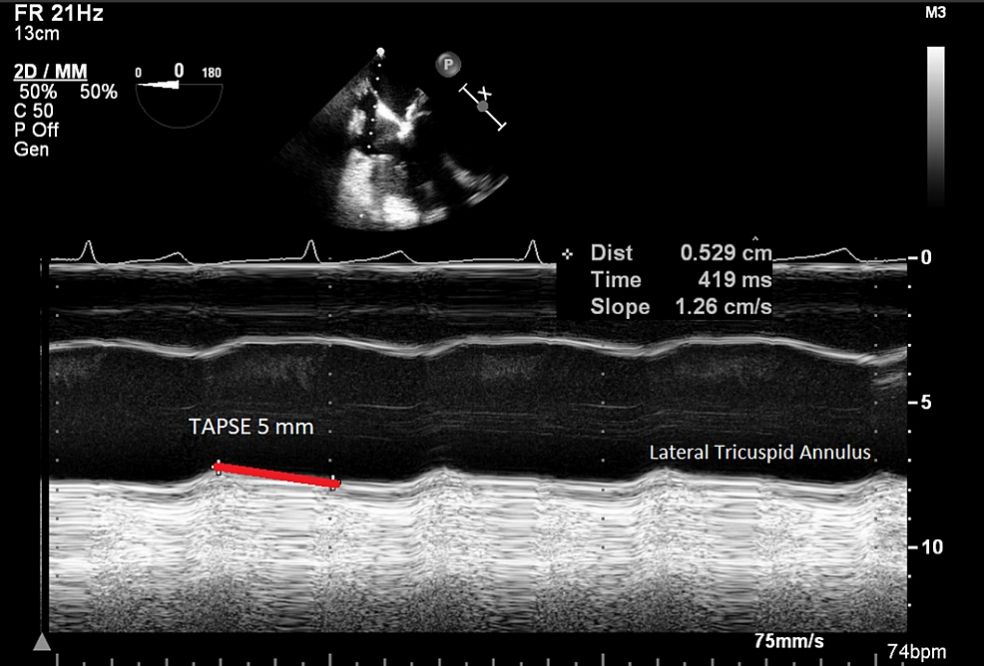
Transoesophageal echo using M-mode to measure Tricuspid Annular Plane Systolic Excursion (TAPSE) (red line) showing severe right ventricular dysfunction.

The surgeon made some adjustments, but the rSO_2_ continued to fall along with a change in medication from GTN to noradrenaline. When the IMAR was removed, the rSO_2_ improved. On cardiopulmonary bypass (CPB), the rSO_2_ was kept above baseline. Grafts were internal mammary artery to the left anterior descending artery and saphenous vein to the circumflex artery. The first attempt to come off CPB on low dose inotropes failed, and he subsequently came off CPB on normal doses of adrenaline (0.05 µg/kg/min), noradrenaline (0.05 µg/kg/min), milrinone (0.23 µg/kg/min), atrial-ventricular sequential pacing and an intra-aortic balloon pump. Figure 3 shows TAPSE reduced to 5mm and indicates global RV dysfunction.

**Figure 4:**
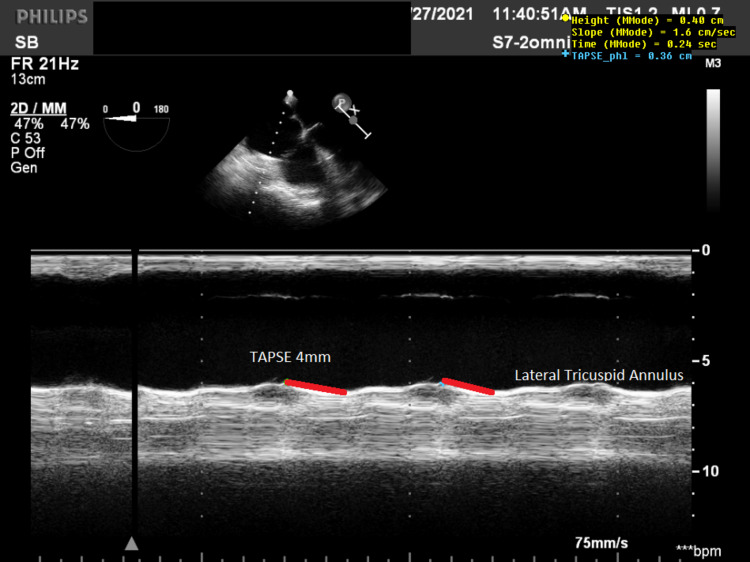
Tricuspid Annular Plane Systolic Excursion (TAPSE) (red line) 4mm on Postoperative Day (POD) 1 shows no Right Ventricle recovery.

In the ICU, his inotropes escalated, plus the addition of vasopressin (0.04µg/kg/min) and nitric oxide (10 ppm). TEE postoperative day (POD) 1 showed a TAPSE of 4 mm (Figure 4).

His blood results from before surgery to POD 14 are shown in Table 1.

**Table 1:** Results and observations during the pre-, intra- and postoperative period. TAPSE: tricuspid annular plane systolic excursion. NSTEMI: non-ST elevation myocardial infarction. POD: postoperative Day. CPB: cardiopulmonary bypass.

	NSTEMI 5 days preop	1 day preoperatively	Intraoperative		POD 1	POD 3/4	POD 12/14
Temperature ^o^C	36.2	36.5	38.1		36.5	36.6	38.2
White Blood Cells (4-11,000 x10^9^/l)	5.4	7.1			25.4	43.6	10.7
Neutophils (2-7.5x10^9^/l)	2.3	6.4			20.4	11.9	8.1
C-reactive protein					231	273	71
Procalcitonin (0.25-2.0µg/l)					160	52	9.1
Lactate (0.7-2.0mmol/l)					12.1	3.2	0.8
TAPSE mm	25mm		11 pre-CPB	5 post-CPB	4	11	13
Troponin I (<14pg/ml)	583				4,245	1,393	318

**Figure 5:**
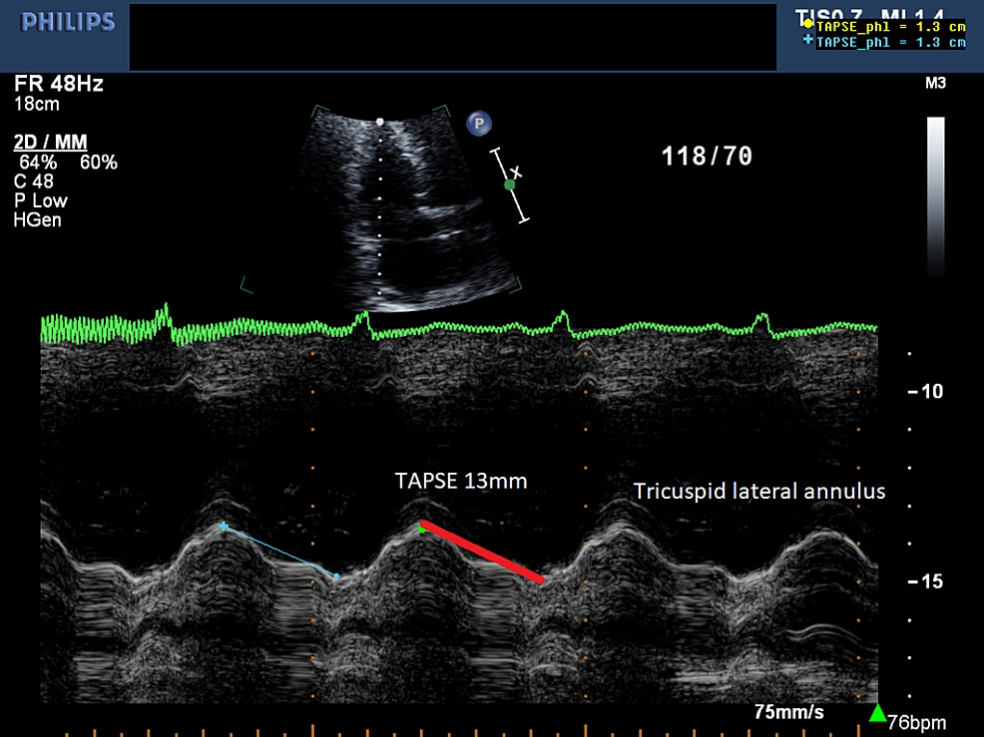
Post-Operative Day 14 the TAPSE is 13mm (red line) showing almost complete right ventricular recovery. TAPSE: tricuspid annular plane systolic excursion.

On POD 1, the WBC and neutrophils had increased significantly along with his troponin I. LV function was preserved, and we suspected RV ischaemia. A POD 1 angiogram showed no occlusion to the right coronary artery. By the POD 3, his haemodynamics stabilised, and blood cultures revealed a Gram-negative organism, *Enterobacter Cloacae*, with sputum positive for *Serratia Marcescens*, both sensitive to meropenem. Over the next five days, all vasoactive support was weaned off, later requiring treatment for his underlying hypertension. Extubation was on POD 12 despite a positive blood culture for *Stenotrophomonas maltophilia* sensitive to moxifloxacin. Rehabilitation was slow, but with normal neurological function, and by POD 14, the TAPSE was 13 mm Figure 5.

## Discussion

Leucocytosis (>11,000 cells/µl) pre-operatively is strongly associated with poor outcomes following cardiac surgery [1]. Our patient did not have a leucocytosis but was incubating a Gram-negative bacteria, which was the cause of septic shock post-operatively. Similarly, there was no pyrexia on his pre-operative check. We could not find any cases in the cardiac literature in which the patient's vital condition changed so much from leaving the ward to arriving in the operating room. However, pyrexia of unknown origin and positive blood cultures for Gram-negative bacilli are at least 26-fold more common in patients on dialysis [2]. With this in mind, when a dialysis patient reports feeling cold and is warm to touch, we should have a low threshold to repeat vital observations such as a temperature as these patients are at higher risk of infection. Even if this had not been detected until after the patient was anaesthetised, consideration must be made as to whether surgery should be postponed.

In addition, our patient suffered RV dysfunction. In the operating room, we tend to think of ventricular dysfunction in terms of ischaemia, however, dysfunction is well described as a result of sepsis [3]. Was the post-CPB RV dysfunction due to ischaemia caused by the compression of the IMAR or inadequate myocardial protection on CPB or secondary to the septicaemia? The timeline suggests compression and subsequent dysfunction caused by the IMAR as evidenced by the requirement for inotropic support and rising CVP prior to CPB.

The post CPB rise in troponin I (higher than after his NSTEMI) and coincident reduction in TAPSE, followed by recovery, could have been either the IMAR or poor myocardial protection on CPB. Both suggest ischaemia or infection rather than significant infarction. Complications caused by the IMAR have been described, with most papers focusing on the sternal circulation rather than acute RV dysfunction [4,5]. Compromised RV function during mammary harvesting is commonly seen. The anesthetist notices an increase in the CVP and a decrease in the mean arterial pressure. Though neither may be dramatic, the resultant reduction in cerebral perfusion pressure can cause a fall in rSO_2_ which can be the first alert, as in our case. A similar scenario is seen during beating heart surgery where the CVP is seen to increase during all coronary anastomosis causing a decrease in cardiac output most marked during anastomosis of the obtuse marginal artery [6]. Typically, the RV recovers when the retractor is removed but not in this case.

Sepsis can cause acute changes in myocardial function; usually, both LV and RV are affected but RV dysfunction is well documented, with recovery over 7-10 days, but a worse one-year survival than patients with no RV involvement [7]. One study of 393 patients with early sepsis found, using TAPSE as a marker, 48% of patients suffered RV dysfunction resulting in a worse outcome than sepsis without RV dysfunction [8]. Even though not all inflammatory cytokines are correlated with myocardial dysfunction in sepsis, troponin I is known to correlate well [9,10]. Our patient’s response to antibiotics and recovery in the first post-operative week imply sepsis was a possible cause for the RV dysfunction.

## Conclusions

We report a patient who presented for urgent CABG surgery following an NSTEMI and renal failure. This put him at high risk for myocardial ischaemia and infection. At the induction of anesthesia in the operating room, he developed pyrexia, hypertension, tachycardia, and hypercapnia. The surgery went ahead, and during the surgery, the patient suffered right ventricular impairment probably from the internal mammary artery retractor but could have been sepsis-related. In ICU, the patient was septicaemic and suffered a right ventricular failure. After 12 days ventilated, he made a full recovery. Even though anesthetised, his infection risk was high, and surgery should have been postponed based on the pyrexia alone. Also, the haemodynamic effect of the IMAR on the RV can cause significant damage to the RV if the compression is prolonged. As such vigilance, and response to rSO_2_ are required. Neither of these two learning points is widely discussed in the literature.
